# Multimodal machine learning in precision health: A scoping review

**DOI:** 10.1038/s41746-022-00712-8

**Published:** 2022-11-07

**Authors:** Adrienne Kline, Hanyin Wang, Yikuan Li, Saya Dennis, Meghan Hutch, Zhenxing Xu, Fei Wang, Feixiong Cheng, Yuan Luo

**Affiliations:** 1 Department of Preventive Medicine, Northwestern University, Chicago, 60201 IL USA; 2 Department of Population Health Sciences, Cornell University, New York, 10065 NY USA; 3 Cleveland Clinic Lerner College of Medicine, Case Western Reserve University, Cleveland, 44195 OH USA

**Keywords:** Health care, Medical research

## Abstract

Machine learning is frequently being leveraged to tackle problems in the health sector including utilization for clinical decision-support. Its use has historically been focused on single modal data. Attempts to improve prediction and mimic the multimodal nature of clinical expert decision-making has been met in the biomedical field of machine learning by fusing disparate data. This review was conducted to summarize the current studies in this field and identify topics ripe for future research. We conducted this review in accordance with the PRISMA extension for Scoping Reviews to characterize multi-modal data fusion in health. Search strings were established and used in databases: PubMed, Google Scholar, and IEEEXplore from 2011 to 2021. A final set of 128 articles were included in the analysis. The most common health areas utilizing multi-modal methods were neurology and oncology. Early fusion was the most common data merging strategy. Notably, there was an improvement in predictive performance when using data fusion. Lacking from the papers were clear clinical deployment strategies, FDA-approval, and analysis of how using multimodal approaches from diverse sub-populations may improve biases and healthcare disparities. These findings provide a summary on multimodal data fusion as applied to health diagnosis/prognosis problems. Few papers compared the outputs of a multimodal approach with a unimodal prediction. However, those that did achieved an average increase of 6.4% in predictive accuracy. Multi-modal machine learning, while more robust in its estimations over unimodal methods, has drawbacks in its scalability and the time-consuming nature of information concatenation.

## Introduction

Clinical decision support has long been an aim for those implementing algorithms and machine learning in the health sphere^1–3^. Examples of algorithmic decision supports utilize lab test values, imaging protocols or clinical (physical exam scores) hallmarks^4,5^. Some health diagnoses can be made on a single lab value or a single threshold, such as in diabetes in older adults^6^. Other diagnoses are based on a constellation of the signs, symptoms, lab values and/or supportive imaging and are referred to as a clinical diagnosis. Oftentimes these clinical diagnoses are based on additive scoring systems that requires an admixture of positive and negative hallmarks prior to confirmatory labeling.

**Fig. 1:**
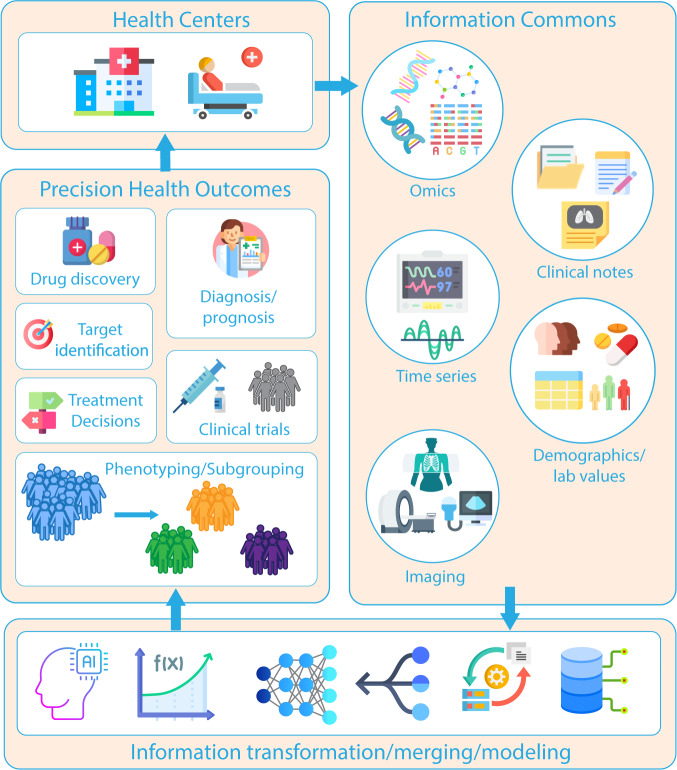
Multimodal precision health; the flow of information. Information moves in a cyclical pattern from health centers to information commons, where it can be transformed and algorithmic modeling performed. These algorithms provide insight into many different health outcomes such as clinical trials, phenotyping, drug discovery, etc. These insights should return to health centers and practitioners to provide the most efficient, evidence-based medicine possible.

The modus operandi of a clinical diagnosis may fail to consider the relative weighting of these disparate data inputs and potentially non-linear relationships highlighting the limitations of human decision-making capacity. The strength of algorithmic decision-making support is that it can be used to offload such tasks, ideally yielding a more successful result. This is the promise of precision medicine. Precision medicine/health aims to create a medical model that customizes healthcare (decisions, treatments, practices etc.) that are tailored to either an individual or patient phenotype^7^. This includes tracking patients’ health trajectories longitudinally^8^, oftentimes incorporating genetics/epigenetics^9,10^ and mathematical modeling^11^ where diagnoses and treatments incorporate this unique information^12^. Contrast this with a one-drug-fits-all model, where there is a single treatment per disorder. Figure 1 illustrates the flow of information from hospitals/care centers that generate disparate data. It is through computational modeling and information fusion that outcomes of interest such as drug and treatment targets ultimately facilitate better decision making at the patient level in those care centers. This phenomenon has sparked an interest in fusion studies using health care data.

Undertakings to characterize this literature have been performed by Huang et al.^13^, who performed a systematic review of deep learning fusion of imaging and EHR data in health. However, it was limited to EHR and imaging data and deep learning applications. A follow-up review article included a commentary on omics and imaging data fusion^14^. The purpose of this study is to highlight the current scope of this research domain, summarize and offer suggestions to advance the field. The current study is more inclusive in the breadth of the types of machine learning protocols used and attempts to encompass all current modalities (information types/sources).

Data fusion is underpinned by information theory and is the mechanism by which disparate data sources are merged to create an information state based on the sources’ complementarity^15,16^ (Box 1). The expectation in machine learning is that data fusion efforts will result in an improvement in predictive power^17,18^ and therefore provide more reliable results in potentially low validity settings^19^. Data fusion touts the advantage that the results of modeling become inherently more robust by relying on a multitude of informational factors rather than a single type. However, the methodology of combinatory information has drawbacks; it adds complexity to specifying the model and reduces the interpretability of results^19,20^.

Data from different sources and file formats are rarely uniform, and this is especially the case with clinical data^21^. For example, data sets can have different naming conventions, units of measure, or represent different local population biases. Care must be taken to search and correct for systematic differences between datasets and assess their degree of inter-operability. For example, Colubri et al. aggregated computed tomography (CT) and PCR lab values, by performing an intra-site normalization. This ensured that the values were comparable across sites. In doing so they discarded several potentially informative clinical variables since they were not all available in all datasets^22^.

A balance is required to allow information that is similar to work together (harmonization) and retain data purity (information correspondence)^23^. Successful fusion uses data harmonization techniques that assure both in the quality control of the integration process. Clinical data harmonization requires multidisciplinary research among medicine, biology, and computer science. The clinical area of heart failure with preserved ejection fraction (HFpEF) saw novel applications of multiple tensor factorization formulations to integrate the deep phenotypic and trans-omic information^24^, and this extends to other areas of precision medicine^25^. To increase the portability of EHR-based phenotype algorithms, the Electronic Medical Records and Genomics (eMERGE) network has adopted common data models (CDMs) and standardized design patterns of the phenotype algorithm logic to integrate EHR data with genomic data and enable generalizability and scalability^26–29^.

There are three main types of data fusion that are used in machine learning; early (data-level), intermediate (joint), and late (decision-level)^30^. In the case of early fusion, multiple data sources are converted to the same information space. This often results in vectorization or numerical conversion from an alternative state, such as that performed by Chen et al. via vectorized pathology reports^31^. Medical images possess characteristics that can undergo numerical conversion based on area, volume, and/or structural calculations^32^. These are then concatenated with additional measurements from structured data sources and fed into an individual classifier. Canonical correlation analysis^33^, non-negative matrix factorization^34,35^, Independent Component Analysis (ICA) and numerical feature conversion methodologies exist as common options to transform all data into the same feature space^36^.

Intermediate data fusion occurs as a stepwise set of models and offers the greatest latitude in model architecture. For example, a 3-stage deep neural learning and fusion model was proposed by Zhou et al.^37^. Stage 1 consists of feature selection by a soft-max classifier for independent modalities. Stages 2 and 3 constitute combining these selected features, establishing a further refined set of features, and feeding these into a Cox-nnet to perform joint latent feature representation for Alzheimer’s diagnosis. In contrast to early fusion, intermediate fusion combines the features that distinguish each type of data to produce a new representation that is more expressive than the separate representations from which it arose.

**Fig. 2:**
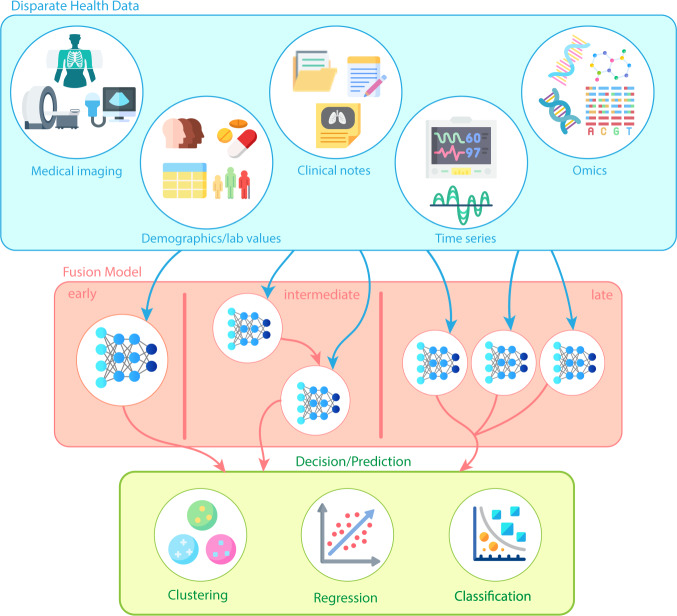
Early, intermediate, and late fusion; flow ofinformation from information commons to model structure to outcomes. Information fusion can occur in a myriad of ways. In machine learning, early, intermediate, and late fusion is typified by if all the information flows into a single model (early), a step-wise fashion where outputs from one model become inputs for another (intermediate), and lastly, where all unique data types undergo separate modelling after which ensembling and/or voting occurs (late).

In late fusion, typically multiple models are trained where each model corresponds to an incoming data source. This is akin to ensemble learning, which offers better performance over individual models^38^. Ensemble methods use multiple learning algorithms (typically applied to the same dataset) to obtain better predictive performance than could be obtained from any of the constituent learning algorithm alone. However, multimodal machine learning ensemble here can refer to ensemble learning within a data type or across data types. These take symbolic representations as sources and combine them to obtain a more accurate decision^39^. Bayesian’s methods are typically employed at this level^40^ to support a voting process between the set of models into a global decision. Within late fusion there has been headway made to perform multitask deep learning^41–47^. A schematic for the 3 subtypes of data fusion is presented in Fig. 2. Attributes in the fusion techniques are shown in Table 1.

**Table 1:** Comparison of fusion techniques.

Attribute	Early	Intermediate/joint	Late/decision
Scalable	No	Yes	Yes
Multiple models needed	No	Yes	Yes
Improved accuracy	Yes	Yes	Yes
Voting of multiple models	No	Yes	Yes
Interaction effects across sources	Yes	Yes	No
Implemented in health	Yes	Yes	Yes

Box 1 Terms and Concepts **Multimodal machine learning:** the area of machine learning concerned with bringing together disparate data sources to capitalize on the unique and complementary information in an algorithmic framework.

**Data harmonization:** using machine learning to unify different data sources to improve its quality and utilization.

**Multiview machine learning:** another term for multimodal machine learning.

**Data fusion:** the specific methodologies undertaken to perform data integration for multimodal/multiview machine learning; they come in three broad categories: early, intermediate/joint, and late.

## Results

### Topic Modeling

**Fig. 3:**
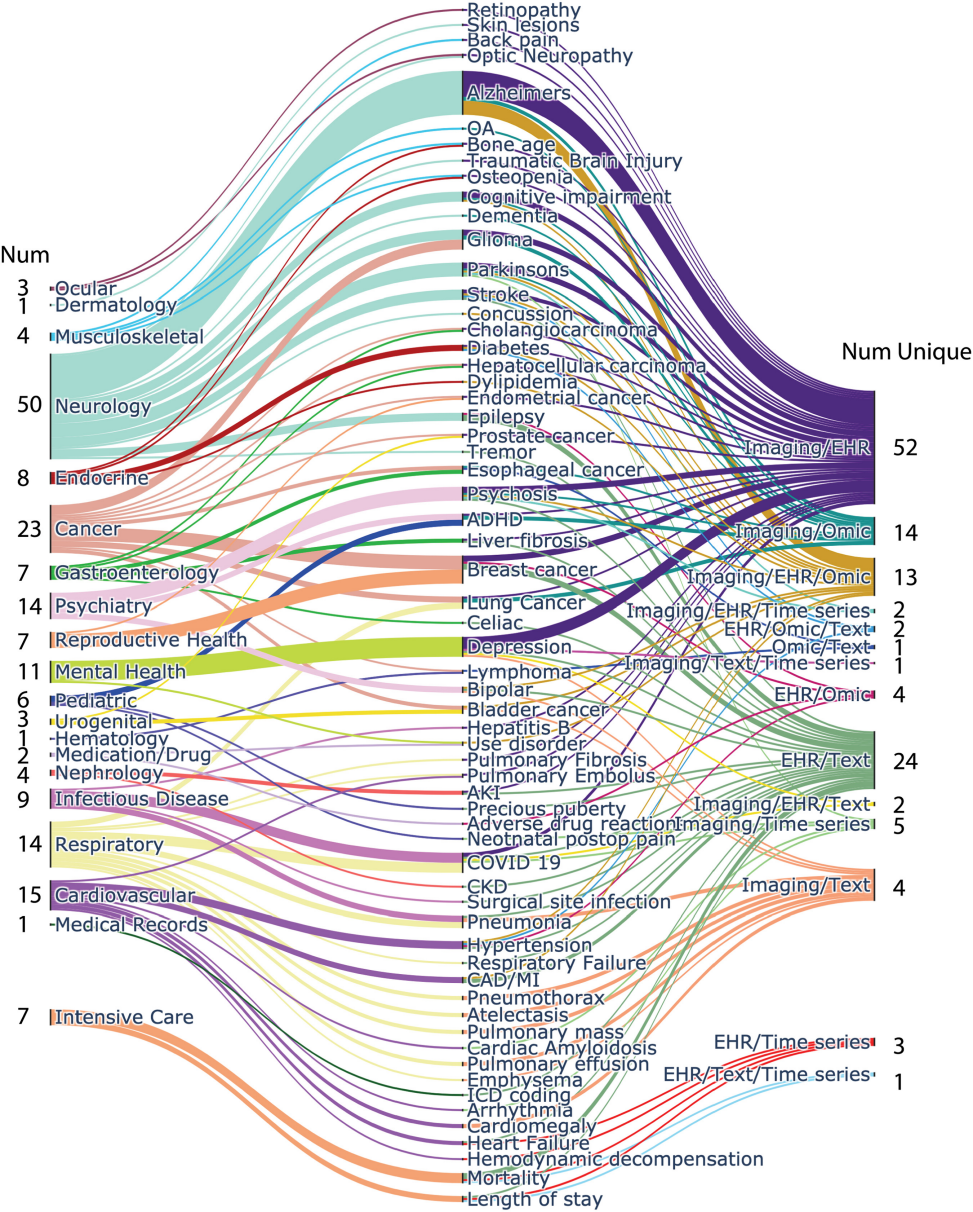
Topic and Modality Modeling. Neurology, and in particular, Alzheimer’s disease investigations accounted for the most papers published on this topic (*n* = 22). With the onset of the COVID-19 pandemic, several primary research articles were dedicated to this topic, which can be arrived at through the respiratory or infectious disease hierarchies. All papers noted in this review used either two or three disparate data sources when fusing their data, and specifically that of imaging and EHR (*n* = 52), was the most prevalent.

**Fig. 4:**
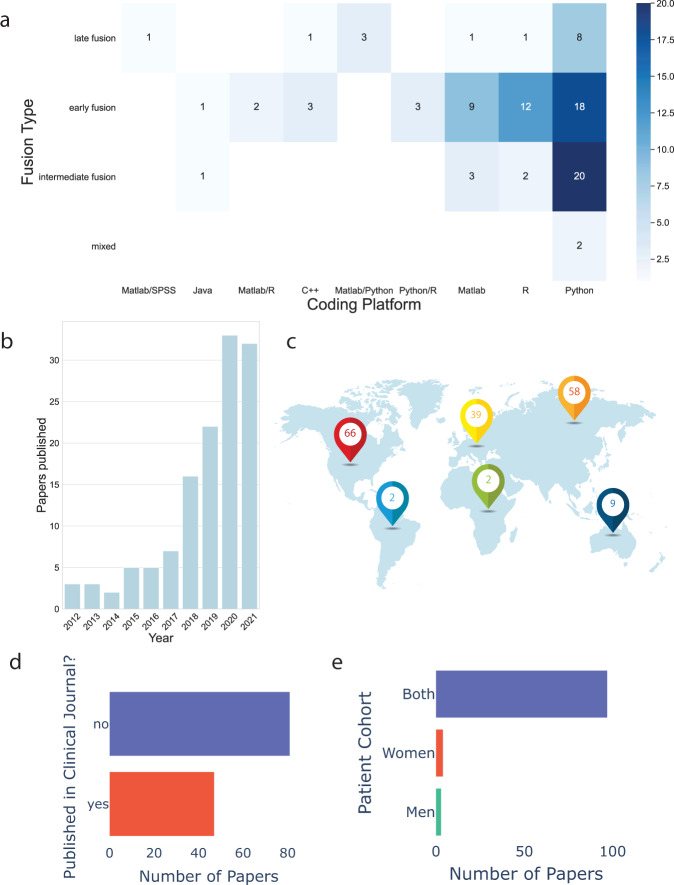
Meta-data from the review process. **a** Heat map of fusion type broken down into the coding platforms papers used by summing over paper counts (those that mentioned platform used), the most popular being the Python platform and early fusion. Of note, 37 of the papers did not explicitly mention a platform. **b** Total number of original research papers published in this sphere in the last 10 years. **c** Continental breakdown of author contributions (note some papers have authors from multiple continents). **d** Breakdown of publication type (clinical/non-clinical journal). Less than half (37.6%) of the papers were published in a journal intended for a clinical audience. **e** Sex breakdown of populations studied. Both men and women were represented in the papers, however, the degree of representation varied within an individual studies.

The topic modeling displayed in Fig. 3 showcases the category, specific health ailment under investigation, and the modality type for the studies included. These were subsequently mapped to the category of the combination of information that were merged to create models for prediction/classification/clustering (Table 2). This plot should serve as a resource to fellow researchers to identify areas that are less frequent, such as dermatology^48^, hematology^49^, medication/drug issues such as alcohol use disorder that may offer new research horizons^50^. Figure 4 identifies coding platforms, publishing trend and location over time, author locations and patient cohorts of the papers included in this review.

**Table 2:** Fusion and machine learning methods included in this review.

Fusion type	Machine learning models/techniques implemented	References
Early	Support Vector Machine	^31–34,36,50,51,53–81^
	Random Forest, Decision Trees	^31,33,34,36,67–71,73–76,78–80,82–91^
	Gaussian model	^36,55,92^
	Bayesian models: Bayesian network, Naïve Bayes	^31,54,71,73–75,80,93^
	Regression: Logistic, Ridge, Cox, LASSO, MARS	^31,51,67–69,77,79,80,87,89,90,94–99^
	Multitask learning	^62^
	iMSF	^100^
	Boosted Models, Adaboost	^68,71–75,79,90^
	Deep Neural Networks: DNN, RNN, CNN, DUN, AutoEncoders	^48,57,61,80,81,91,97,101–107^
	Natural Language Processing, GPT, BERT	^81,102,105^
	Clustering: K-means, hierarchical, KNN	^65,73,75,76,100,108^
	Graph models	^107–109^
	Ensemble learning	^65,88^
	Artificial Neural Networks	^57,64,69,73,78,81,88,91,101,104,107,110,111^
	Markov Model	^108^
	Transfer learning	^48,62,97,105,112^
	Multitask learning	^62^
Intermediate	Regression: logistic, multivariate, support vector, LASSO	^41,43,47,59,113–122^
	Support Vector Machine	^37,59,113–115,117,120,123–126^
	Decision Trees, Random Forest	^41,49,59,115–117,120,122,127,128^
	Deep Learning: CNN, DNN, RNN, AutoEncoders	^37,41–47,59,115–117,121–135^
	Boosted Learning: gradient boosting, XGBoost, Adaboost	^116,118,126^
	Natural Language Processing	^122,126,134,136^
	Artificial Neural Network	^43–45,49,116,117,121,126,135^
	Naïve Bayes	^59^
	Ensemble Learning	^120^
	Transfer Learning	^46^
	Multitask learning	^47,137^
	Graph Learning	^136^
Late	Support Vector Machine	^138–143^
	Random Forest, Decision Trees	^130,140–147^
	Regression: LASSO, Logistic, Multivariate, GAM	^141,143,147,148^
	Deep Learning: CNN, DNN, RNN, AutoEncoders	^39,130,140,142,144–146,149–153^
	Ensemble learning	^154^
	Multitask learning	^43,47,59,113,120^
	XGBoost	^143,147,153^
	Artificial neural network	^39,153,155^
	Natural Language Processing	^145,153,155^
	Regression: GLM, Logistic	^140–143,147,155,156^
	Graph network	^157^
	Clustering: KNN, graphs	^144,145,157^
Mixed	Random Forest	^158^
	Artificial neural Network, ElasticNet	^159^

*CNN* convolutional neural network, *DUN* deep unified networks, *MARS* Multivariate adaptive regression splines, *MLP* multilayer perceptron, *GLM* Generalized Linear Model, *DNN* deep neural network, *GAM* generalized additive model, *iMSF* incomplete Multi-Source Feature, *RNN* Recurrent Neural Network.

### Model validation, techniques, and modalities used

Of the models used in the papers, 126/128 explicitly reported performing a validation procedure of them. The most common validation processes performed were N-fold cross validation (55)^51,52^, train test split (51), leave one out cross validation (10), and external dataset (10). A cornucopia of machine learning techniques and methods were used within and across articles in this review. They have been summarized in Table 2, noting in which fusion umbrella subtype they were implemented.

### Early fusion

Most papers were published using early fusion. Of those, most were published using medical imaging and EHR data^34,36,48,53,56,57,60,62–64,68,71,73,75,85–88,90,92–96,98,100,104,111^. Nearly all these papers performed numericalization of image features in essence converting them to structured data prior to processing, however, two performed matrix factorization^34,36^. A combination of EHR and text data was noted in 15 papers^31,54,69,72,79–81,91,99,102,106,112^. Meng et al. created a Bidirectional Representation Learning model used latent Dirichlet allocation (LDA) on clinical notes^112^. Cohen et al. used unigrams and bigrams in conjunction with medication usage^54^. Zeng et al. used concept identifiers from text as input features^81^. Nine papers used early fusion with imaging, EHR and genomic data^32,50,51,55,61,65,83,89,108^. Doan et al. concatenated components derived from images with polygenic risk scores^83^. Lin et al. also created aggregated scores from MRI, cerebral spinal fluid, and genetic information and brought them together into a single cohesive extreme learning machine to predict mild cognitive impairment^55^. Tremblay et al. used a multivariate adaptive regression spline (MARS) after normalizing, removing highly correlated features^89^. Ten papers performed fusion using imaging and genomic data^33,52,70,76–78,82,84,97,110^. Three of these generated correlation matrices as features by vectorizing imaging parameters and correlating them with single nucleotide polymorphisms (SNPs) prior to feeding into the model^33,70,78^. Three papers in this category used EHR and time series^58,74,101^. Both Hernandez and Canniére et al. implemented their methods for purposes of cardiac rehabilitation and harnessed the power of support vector machines (SVMs). However, Hernandez preserved time series information by assembling ECG data into tensors that preserve the structural and temporal relationships inherent in the feature space^74^, while Canniére performed dimensionality reduction of the time series information using t-SNE plots^58^. Two papers comprised early fusion using imaging and time series^67,103^. There were two papers that leveraged EHR and genomic information^66,119^. Luo et al. implemented hybrid non-negative matrix factorization (HNMF) to find coherence between phenotypes and genotypes in those suffering from hypertension^119^. One paper leveraged early fusion using imaging and text data^105^ and another used EHR, Genomics, Transcriptomics, and Insurance Claims^157^.

### Intermediate fusion

Intermediate fusion had the second highest number of papers published. 14 used imaging and EHR data^43,59,113,114,118,121,123,125,126,129,131–133,135,137^. Zihni et al. merged the output from a Multilayer Perceptron (MLP) for modeling clinical data and convolutional neural network (CNN) for modeling imaging data into a single full connected final layer to predict stroke^135^. A very similar approach was taken by Tang et al. who used three-dimensional CNNs and merged the layers in the last layer^113^. EHR and text data were fused together in 11 papers^41,44,80,107,109,116,122,126,134,136,142^. Of these, six^41,44,80,122,134,142^ used long term short term (LSTM) networks, CNNs, or knowledge-guided CNNs^160^ in their fusion of EHR and clinical notes. Chowdhury et al. used graph neural networks and autoencoders to learn meta-embeddings from structured lab test results and clinical notes^107,109^. Pivovarov et al. learned probabilistic phenotypes from clinical notes and medication/lab orders (EHR) data^136^. Two models each employing LDA where data type was treated as a bag of elements and to bring coherence between the two models to identify unique phenotypes. Ye et al. and Shin et al. used concept identifiers via NLP and bag-of-words techniques, respectively, prior to testing a multitude of secondary models^116,126^. In general, clinical notes can provide complementary information to structured EHR data, where natural language processing (NLP) is often needed to extract such information^161–163^. A few studies were published using imaging and genomic^37,117,120^. Here radiogenomics were used to diagnose attention-deficit/hyperactivity disorder (ADHD), glioblastoma survival, and dementia respectively. Polygenic risk scores were combined with MRI by Yoo et al. who used an ensemble of random forests for ADHD diagnosis^120^. Zhou et al. fused SNPs information together with MRI and positron emission tomography (PET) for dementia diagnosis by learning latent representations (i.e., high-level features) for each modality independently. Subsequently learning joint latent feature representations for each pair of modality combination and then learning the diagnostic labels by fusing the learned joint latent feature representations from the second stage was carried out^37^. Wijethilake used MRI and gene expression profiling, performing recursive feature elimination prior to merging into multiple models SVM, linear regression, and artificial neural network (ANN). The linear regression model outperformed the other two merged models and any single modality^117^. Wang et al. and Zhang et al. showcased their work in merging imaging and text information^45,46^. Both used LSTM for language modeling a CNN to generate embeddings that were joined together in a dual-attention model. This is achieved by computing a context vector with attended information preserved for each modality resulting in joint learning. Seldom were articles published using: Imaging/EHR/Text^115^, Genomic/Text^49^, Imaging/Time series^127^, Imaging/Text/Time series^47^, Imaging/EHR/Genomic^130^, Imaging/EHR/Time series^124^, EHR/Genomic^128^, EHR/Text/Time series^42^.

### Late fusion

A much smaller number (*n* = 20) of papers used late fusion. Seven of those used imaging and EHR data types^138,139,144,150,151,154,164^. Both Xiong et al. and Yin et al. fed outputs into a CNN to provide a final weighting and decision^150,151^. Three papers were published using a trimodal approach: imaging, EHR and genomic^130,147,148^. Xu et al. and Faris et al. published papers using EHR and text data^146,155^. Faris et al. processed clinical notes using TF-IDF, hashing vectorizer and document embeddings in conjunction with binarized clinical data^155^. Logistic Regression (LR), Random Forest (RF), Stochastic Gradient Descent Classifier (SGD Classifier), and a Multilayer Perceptron (MLP) were applied to both sets of data independently and final outputs of the two models were combined using different schemes: ranking, summation, and multiplication. Two articles were published using imaging and time series^149,152^ both of which employed CNNs, one in video information of neonates^149^ and the other in chest x-rays^152^. However, they differed in their processing of the time series data. Salekin used a bidirectional CNN and Nishimori used a one-dimensional CNN. Far fewer papers were published using Imaging/EHR/Text^153^, EHR/Genomic/Text^145^, imaging/EHR, time series/^141^, Imaging/Genomic^156^, EHR/Genomic^140^, and Imaging/Text^39^.

### Mixed fusion

Two papers performed multiple data fusion architectures^158,159^. Huang et al. created seven different fusion architectures. These included, early, joint, and late fusion. The architecture that performed the best was the late elastic average fusion for the diagnosis of pulmonary embolism using computed tomography and EHR data^159^. Their Late Elastic Average Fusion leveraged an ElasticNet (linear regression with combined L1 and L2 priors that act as regularizers) for EHR variables. El-Sappagh et al. performed early and late fusion to create an interpretable Alzheimer’s diagnosis and progression detection model^158^. Their best performing model was one that implemented instance-based explanations of the random forest classifier by using the SHapley Additive exPlanations (SHAP) feature attribution. Despite using clinical, genomic, and imaging data, the most influential feature was found to be the Mini-Mental State Examination.

### Clinical relevance

Data fusion may help address sex representation and increase population diversity issues (including minority populations) in health modeling by creating a more representative dataset if one datatype contained more of one sex and another datatype contained more of the other. This reciprocal compensation ability of employing various data sets would also hold true for racial or ethnic diversities.

Less than half (37.6%) of the papers were published in a journal intended for a clinical audience. None of the papers included in the final cohort of studies had created tools for clinical use that had FDA approval. Based on the rising number of papers in this field there is a growing and global need and interest to characterize these findings.

## Discussion

Returning to our research questions, we outlined from the inception of this work, we arrive at Table 3.

**Table 3:** Research questions as outlined in Methods.

RQ1	• The literature published in this area as displayed and characterized in the Results’ section is one that is of growing and global interest. • Fueled by a desire to improve predictive capabilities, relying on complementary and correlative (reinforcing) data. This was found to be the case in the papers surveyed and included in this review, with an increase in 6.4% accuracy. • Most common health topics were neurology and cancer. This is likely fostered by curated databases that lend themselves to multi-modality predictions such as Alzheimer’s Disease Neuroimaging Initiative^165^ and The Cancer Genome Atlas Program^166^. • Dominance of early data fusion methods likely owe their pervasiveness for three reasons: ◦ 2 modalities over 3 means less work overall in model building and deployment. ◦ EHR and image data do not require extensive digital conversion for models as does text. ◦ Early fusion is built on a single model with a multitude of feature inputs and is typically less computationally complex than is intermediate or late fusion. • Seldomly did articles perform comparisons of machine learning findings against their human clinician counterparts. • Several did perform comparisons between uni-modal and multi-modal predictions, with the majority having found a consistent improvement in classification accuracy, sensitivity, and specificity^53,113,159^ when leveraging multi-modal data. • Performance benefits seemingly not limited to a particular subtype of multi-modal strategy that was detectable in our metadata. • Genera recommendation that multi-modal data integration be attempted to improve performance and better mirror a human expert by creating a higher validity environment from which to make clinical decisions.
RQ2	• The analysis techniques are varied and currently do not showcase a gold standard machine learning method in the field. This is likely linked to it being a relatively new and emerging field. • The varied techniques implemented are highlighted in Table 3. • N-cross fold validation was the most common and a robust estimator in the face of bias within a dataset. • Strength of generalizability stems from either the dataset set containing multi-site/location patient data to begin with, or using an external dataset from a remote location^167^.
RQ3	• Health contexts predominantly impacted by this include Neurology and Cancer. • No domain/method laid claim to building translation models via FDA (or equivalent) approval for use in clinical circumstances. • Compare models more readily to physician decision makers^168–171^. This will guide the validity of the environments suitable to machine learning and increase adoption and permit FDA approval of these tools^172^.

Many issues were raised in the papers included in this review. The most common reported limitations were cohorts from a single site, small sample sizes, retrospective data, imbalanced samples, handling of missing data, feature engineering, controlling for confounding factors, and interpretation of the models employed. Samples were most often built from a single hospital or academic medical center^148^. Small sample sizes often lead to poor model fitting and generalizability. The median number of unique patients reported across the studies was 658 with a standard deviation of 42,600. This suggests that while some studies were able to leverage large and multi-center cohorts, a great many were not able to do so^70,82,120,131^.

**Fig. 5:**
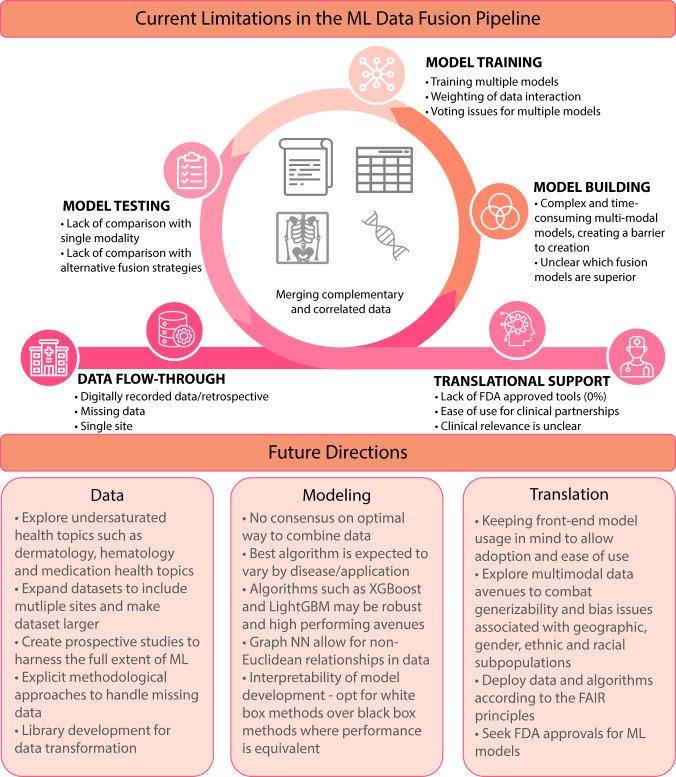
Limitations to multimodal fusion in health and proposed future directions of the fields. Limitations to multimodal fusion implementation are stratified by their location in the workflow. These include issues associated with the underlying data, the modeling that arises from that data, and finally how these are ported back to health systems to provide translational decision support.

Seldom were machine learning investigations on prospective data, an issue endemic in the field^84^. Sample imbalances were often ignored, which results in biased models and misleading performance metrics^75,151^. Missing data were usually ignored by dropping data or imputing, if not dealt with appropriately can skew the results^68,106,173^. More studies need to discuss frequencies and types of missing data^174–177^. Comparison of different imputation methods on the final results should be part of the reporting process^178^. When performing statistical analysis, researchers usually ignored possible confounding factors such as age or gender. Doing so may have major effects on the impact of results^153^. Such possible confounding effects should either be taken into consideration by the model^179,180^ or adjusted for first, prior to reporting model results. Reasonable interpretations of the model and outputs must be presented so that clinicians find the results credible and then use them to provide guidance for treatments. However, most authors did not take the time to interpret the models for clinical audiences. Additionally, how the results may function as a clinical decision support tool. Different types of models warrant different explanations^129,130^. These limitations are highlighted where they occur in the data processing and modeling building pipeline in Fig. 5.

To expedite and facilitate this field, we have outlined several gaps for future research in this field. These are listed in Fig. 5 and explored. Medication/drug topics present an underrepresented area, with only two papers being published in this field^50,66^. Awareness of drug interaction effects is a difficult and growing issue^181–184^, particularly in geriatrics, which gave rise to Beer’s criteria^185^. Performing multimodal machine learning may offer an earlier detection of adverse events associated with medication misuse that is a result of iatrogenic error, non-compliance, or addiction. Similar justifications as outlined above could be applied to other areas seen as ‘under saturated’ such as hematology with only one paper^49^ and nephrology having just three^41,87,99^.

Augmenting clinical decision-making with ML to improve clinical research and outcomes offers positive impacts that have economic, ethical, and moral ramifications, as it can reduce suffering and save human lives. Multiple studies have now pointed out that if the data an ML model is trained on is biased this often yields bias in the predictions^186,187^. Ensuring multisite, representative data will limit model biases. We also advocate for the creation of open access pipelines/libraries to speed up data conversion to make the technology more widely available^188,189^. Improving accuracy at the expense of complex and time-consuming data transformations may mean the predictive power gained from a multimodal approach is offset by this front-end bottleneck, meaning predictions are no longer temporally relevant or useful.

While incorporating disparate data does lend itself to seemingly better predictions^139^, as knowledge around certain diseases accumulates, data fusion in healthcare is an evolving target that warrants proactively adapting to the dynamic landscape^190^. There is no single ML model with ubiquitous applicability. For example, it has been shown in protein-protein interactions that utilization of the XGBoost ensemble algorithm reduces noisy features, maintains the significant raw features, and prevents overfitting^122^. Similarly, LightGBM^191^ has the advantages of faster training speed, higher efficiency, lower memory usage, better accuracy^192^, and has been consistently outperforming other models^193,194^. Graph neural networks can synthesize new connections leading to drug discovery/targets^122^.

In the same vein, models that permit interpretability should always be considered. For example, the Perotte et al.^99^ model was not compared with conventional simpler machine learning classifiers, and collective matrix factorization becomes inherently difficult to interpret^79^. Contrast this with the work of Fraccaro et al. whose study of macular degeneration noted their white box performed as well as black box methods implementions^68^.

As this field and the datasets associated mature there is work needed to address the tenets of data management: Findability, Accessibility, Interoperability, and Reuse of digital datasets (FAIR)^195^. This entails having metadata that are unique/de-identified and searchable, with open or federated access points (Findability/Accessibility), data that are shared broadly (Interoperable), and finally data that contain accurate and relevant attributes under a clear data usage agreement/license (Reusable). It is imperative there exist a clear definition of outcomes, assessment of biases and interpretability/transparency of results, and limitations inherent in its predictions^196^.

Of crucial importance for uptake is that predictions be patient-specific and actionable at a granular level^197^. For example, a 30-day readmission prediction algorithm^106^, if implemented, may inform resource management and prompt additional research that may decrease the number of patients re-admitted. Linden et al. developed Deep personalized LOngitudinal convolutional RIsk model (DeepLORI) capable of creating predictions that can be interpreted on the level of individual patients^122^. Leveraging both and clinical and empirically driven information to create meaningful and usable recommendations^136^ may improve clinician/end-user under understanding by relating to existing frameworks. Resources such as CRISP-ML provide a framework for moving use cases into more practical applications^198^, while efforts to vie for Food and drug administration (FDA) approvals as a tool for use are encouraged to increased adoption.

Deployment of models with user interfaces annotating limitations inherent on those predictions^196^ will allow clinical decision makers to interface and implement change accordingly. Follow-through on the aforementioned tasks will push individual fields to create recommendations for subsequent real-world implementations that are relevant, actionable, and transcend regional/subpopulation differences. Limitations of this scoping review include that it is not a systematic review. Therefore, it is possible that some titles that should have been included were missed. As the primary purpose of this study was to perform scientific paper profiling on multimodal machine learning in health, a critical appraisal of individual methodological quality of the included studies was not performed. However, commentary is provided on the methodological limitations that could have affected their results and impacted their claims. This review offers comprehensive meta-data and evaluation across health domains, immaterial to the type of machine learning or the data used. This work serves as both a summary and steppingstone for future research in this field. Data fusion in health is a growing field of global interest. The topic areas of health that have high frequency relative to others were neurology and cancer, which serve to highlight opportunities for further exploration in understudied topics (hematology, dermatology). Unimodal machine learning is inherently in contrast to current routine clinical practice in which imaging, clinical or genomic data are interpreted in unison to inform accurate diagnosis and warrants further work for ease of use and implementation. Overall, it appears justified to claim that multi-modal data fusion increases predictive performance over unimodal approaches (6.4% mean improvement in AUC) and is warranted where applicable. Multimodal machine learning may be a tool leveraged in precision medicine to further subgroup patients’ and their unique health fingerprint. Furthermore, as no papers in our review sought FDA approval, we advocate for more efforts into model translation and explore necessities that facilitate that end.

A dashboard resource published in conjunction with this review article is available at: https://multimodal-ml-health.herokuapp.com/. This dashboard was created as an interactive infographic-based display of the major findings presented in this paper. To foster future work, a drop-down menu was created to help researchers filter the underlying data file of titles based on the specific overarching health topic by selection. This will facilitate the location of relevant papers.

## Methods

### Search strategy and selection criteria

Inclusion requirements were: (a) original research article; (b) published within the last 10 years (encompassing years 2011–2021); (c) published in English; and (d) on the topic of multi-modal or multi-view using machine learning in health for diagnostic or prognostication applications.’Multi-modal’ or’multi-view’ for our context means the multiple data sources were not of the same type. For example, while a paper using CT and MRI may be considered multi-modal imaging; however, under our criteria it would be considered uni-modal (i.e., only included imaging). Exclusions for the purposes of this review were: (a) scientific articles not published in English; (b) commentaries or editorials; or (c) other review articles. Papers were also excluded if the data were not human-derived. We also excluded papers where the fusion already occurred at the data generation stage, such as spatial transcriptomics producing integrated tissue imaging and transcriptomics data^199–201^. All papers underwent a 2-person verification for inclusion in the manuscript.

**Fig. 6:**
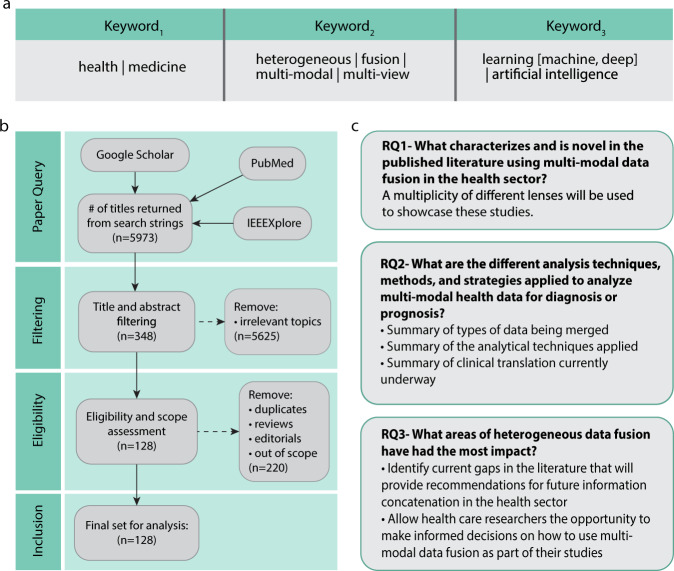
Overview of our PRIMSA-SCR process. **a** Health-related keyword, Multimodal-related keyword, machine learning-related keywords, |: or. For example, “health + heterogeneous data + machine learning” would be one of the search strings. **b** Overview of study inclusion process. **c** Research questions posed.

Search strings were established via literature searches and domain expertize. Additional keywords were identified based on key word co-occurrence matrices established from the abstracts of the previously included articles. Figure 6a displays the search strings, where an individual string would include one keyword from each column, this was performed for all combinations of search strings. An overview of the inclusion/exclusion process is noted in Fig. 6b and follows the standard set by PRISMA extension for scoping reviews^202^.

### Data extracted

Information garnered from the articles included title, year published, FDA approval of the tool, whether published in a clinical journal, author affiliations, number of authors, locations (continents), and abstract. Health topic(s) addressed were extracted, as well as the broader medical topic(s) that encompass the disease. For example, lung cancer would be the specific disease in question. It arises from the topics of Cancer and Respiratory according to our classification. Health topic classification was overseen and reviewed by a medical doctor to ensure accuracy. As multiple health topics often encompassed a single health disease addressed in each paper, several papers are counted twice. This is true when being mapped from the right side of the Sankey plot to the specific health disease in the middle.

We recorded and extracted the number of different modalities and the divisions (i.e., text/image vs EHR/genomic/time series) used. The objective of each paper was extracted in a 1–2 sentence summary along with the keyword (if available). Patient characterization in the studies was performed by ascertaining the number of unique patients in the cohort and patient sex (i.e., Men/women/both or not mentioned).

Computational information extracted included: (a) the coding interface(s) used in data processing/analysis, (b) machine learning type, (c) data merging technique (early, intermediate, late), and (d) types of machine learning algorithms used. Whether validation was performed (yes/no), the statistical tests run, the nature of the validation, and outcomes measures were all recorded for each paper. The significance, impact, and limitations of each paper were extracted by reviewing the primary findings and limitations as noted in the papers.

### Reporting summary

Further information on research design is available in the Nature Research Reporting Summary linked to this article.

## Supplementary information

Reporting Summary
